# A tunable high-pass filter for simple and inexpensive size-segregation of sub-10-nm nanoparticles

**DOI:** 10.1038/srep45678

**Published:** 2017-04-27

**Authors:** N. C. Surawski, S. Bezantakos, K. Barmpounis, M. C. Dallaston, A. Schmidt-Ott, G. Biskos

**Affiliations:** 1 Energy, Environment and Water Research Center, The Cyprus Institute, Nicosia, 2121, Cyprus; 2 Faculty of Applied Sciences, Delft University of Technology, Delft, 2628-CN, The Netherlands; 3 Department of Chemical Engineering, Imperial College London, London, SW7 2AZ, United Kingdom

## Abstract

Recent advanced in the fields of nanotechnology and atmospheric sciences underline the increasing need for sizing sub-10-nm aerosol particles in a simple yet efficient way. In this article, we develop, experimentally test and model the performance of a High-Pass Electrical Mobility Filter (HP-EMF) that can be used for sizing nanoparticles suspended in gaseous media. Experimental measurements of the penetration of nanoparticles having diameters down to ca 1nm through the HP-EMF are compared with predictions by an analytic, a semi-empirical and a numerical model. The results show that the HP-EMF effectively filters nanoparticles below a threshold diameter with an extremely high level of sizing performance, while it is easier to use compared to existing nanoparticle sizing techniques through design simplifications. What is more, the HP-EMF is an inexpensive and compact tool, making it an enabling technology for a variety of applications ranging from nanomaterial synthesis to distributed monitoring of atmospheric nanoparticles.

Size-segregating nanoparticles is important for a variety of nanotechnology and science applications. Employing nanoparticles that have diameters of or below a given size, for instance, can attribute specific properties to materials and devices developed in diverse disciplines such as catalysis^1^, sensing^2^, fluidics^3^ and delivery of nanomedicine^4^, amongst many others. In addition, measuring the size of nanoparticles in the atmospheric environment can help us understand their effect upon human health^5^,^6^ and climate^7^. Interestingly, recent research efforts in aerosol metrology have focussed on the development of low-cost, portable and easy-to-use nanoparticle sizers that can be deployed for a variety of environmental monitoring purposes^8^,^9^. In this article, we develop, experimentally test and model a High-Pass Electrical Mobility Filter (HP-EMF; high-pass with respect to electrical mobility diameter) that has many advantages compared to existing systems for determining the size of gas-suspended nanoparticles.

An effective and commonly used way to size aerosol nanoparticles is by determining their electrical mobility with a Differential Mobility Analyser (DMA)^10^. DMAs employ a well defined electrostatic field, established between two electrodes, that acts perpendicularly to the direction of the flow carrying the nanoparticles. Charged nanoparticles are introduced through a narrow slit near the outer electrode and a nanoparticle free sheath air advects them along the classification zone of the DMA. Charged nanoparticles of one polarity (positive or negative depending on the direction of the electric field) are deflected towards the opposite electrode where those having electrical mobilities, and hence sizes, within a very narrow range (i.e. nearly monodisperse) exit as the outlet flow of the instrument. Nanoparticle trajectories within the classification zone of the DMA are shown in Fig. 1a, demonstrating that only a narrow bandwidth of nanoparticle mobilities (red curve) are transmitted at a fixed electrode voltage, with the remainder depositing upstream of the outlet slit on the inner electrode, or being carried out with the excess flow of the instrument (blue curves). Because a narrow bandwidth of nanoparticle mobilities are transmitted at a fixed electrode voltage (cf. Fig. 1b), the DMA can be thought of as a band-pass filter^11^. This is the primary reason that it is a widely used nanoparticle sizing tool in laboratory and field investigations. Despite that, however, the high manufacturing cost and weight of the DMA prevent it from being widely deployed for applications that require portability and a fine grid of measurements. Although recent advances in fabrication techniques^12^ have begun to address the cost and weight issues associated with traditional DMAs, the systems required for its operation (e.g. sheath flow control) make it a bulky and complex system to use on a routine basis.

Another approach for sizing aerosol nanoparticles was offered by the Electrical Aerosol Analyser (EAA)^13^, which has now been discontinued commercially. The EAA also relied on perpendicular flow and electrostatic fields, but in contrast to the DMA it transmitted particles having diameters above a given threshold value (defined by the operating conditions of the instrument). As a result, it can be thought of as a high-pass filter with respect to the transmitted electrical mobility diameter (Fig. 1c,d). A simpler alternative to size-segregate nanoparticles is with an electrostatic precipitator (EP)^14^. As shown in Fig. 1e charged nanoparticles passing through an EP are deposited by an electrostatic field that acts perpendicularly to the aerosol flow, and unlike the DMA or the EAA, it does not require a sheath flow. Because the EP transmits nanoparticles having diameters above a threshold value, it can also be thought of as a high-pass filter (cf. Fig. 1f). A disadvantage associated with EPs, however, is their poor nanoparticle sizing performance, as reflected by the poor sharpness of the associated penetration curves.

In contrast to the EP, the HP-EMF employs an electric field parallel to the aerosol flow, which can be achieved by applying a potential difference between the inlet and an intermediate position along it. A schematic of the HP-EMF indicating its principle of operation is shown in Fig. 2. Incoming charged nanoparticles of the same polarity as the high-voltage applied to the HP-EMF are initially decelerated (Zone I) and then accelerated (Zone III) as they move along the filter. The repelling force in the decelerating zone increases the residence time for nanoparticles within it, which increases the opportunity for both diffusional and electrostatic deposition to the walls of the HP-EMF. A key benefit of the combined effect of both of these processes (i.e. electrostatic and diffusional deposition), which is expected to become pronounced with decreasing nanoparticle diameters, is that it gives rise to improved size-segregating ability (SSA; i.e. a metric that quantifies sizing performance; see discussion below and in the Supplementary Information) compared to conventional EPs, especially for nanoparticles smaller than 10 nm. This makes the HP-EMF a highly suited tool for sizing nanoparticles in this size range compared to other approaches. In addition, the HP-EMF does not require a sheath flow, which simplifies its ease of operation compared to DMAs.

The HP-EMF shares design features similar with devices previously presented for size-segregating nanoparticles. It is worth noting here that the theory of sizing sub-10-nm aerosol nanoparticles shares a lot in common with the field of plasma chromatography^15^. The introduction of an electric field parallel, or oriented axially, to the flow in aerosol particles sizers has been pursued by Schlatter *et al*.^16^, Loscertales^17^ and Oberreit^18^. The HP-EMF, however, offers design simplifications through not requiring a sheath flow or counterflow and is also a compact tool unlike previous devices.

## Results and Discussion

### Performance of the High-Pass Electrical Mobility Filter

Figure 3 shows the penetration of nanoparticles through the HP-EMF as a function of their size. Evidently, nanoparticles smaller than a threshold diameter are not transmitted for specific electrical potential differences between the inlet and the intermediate position at which the high voltage is applied. The sizing and filtration performance of the HP-EMF and its agreement with the analytic model provided recently by Tammet^19^ is also shown in Fig. 3. According to this model, relative penetration *P*_*r*_ is given by:


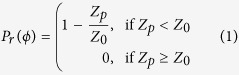


where *Z*_*p*_ is the electrical mobility of any incoming nanoparticle and *Z*_0_ is the threshold mobility determined as the ratio of the average flow velocity in the HP-EMF 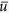 and the mean electric field strength 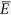 in the decelerating segment (i.e. 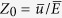). A first approximation of the electric field strength is 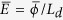, where 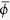 is the mean potential in the decelerating segment along the axis of symmetry and *L*_*d*_ is the decelerating segment length. Alternatively, the electric potential *ϕ* based on solving Laplace’s equation (see Supplementary Information for the derivation) is given by:


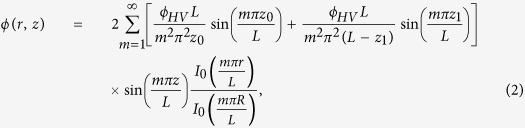


where *L* and *R* are the length and radius of the HP-EMF, respectively, *r* and *z* denote radial and axial position along the HP-EMF, respectively, *ϕ*_*HV*_ is the electric potential applied to the surface of the HP-EMF in the high voltage zone (i.e. zone II), *z*_0_ and *z*_1_ denote the start and end of the high voltage zone, respectively, *I*_0_ denotes a modified Bessel function of the first kind and *m* is an integer.

Agreement between measurements and predictions using the analytic model match within experimental uncertainty (solid lines in Fig. 3) only after adjusting the mean potential (calculated along the axis of symmetry in Zone I) in the decelerating segment to 58.1% of that applied to the surface of the HP-EMF for all tested voltage settings. For example, when a 1000 V potential was applied to the surface of the HP-EMF at the intermediate position, the fitted potential (estimated from a quasi-Newton non-linear least squares algorithm) was estimated to be 581 V in the decelerating segment. Alternatively, the mean potential was also calculated analytically by Equation 2 (dashed lines in Fig. 3). This led to a mean absolute deviation in penetration (across all voltage settings) between the measurements and predictions using the analytic model of only 5.0%, which is well within the nominal uncertainties of the measurement system (~15%). Even though agreement between measurements and the analytic model is excellent, other modelling approaches that either partially or fully capture the physical principles underpinning the HP-EMF are discussed later.

The HP-EMF can be employed as a nanoparticle sizer if the relation between penetration and electrical mobility diameter is known. Experimentally, these curves are created through successive differential measurements of particle number concentration with and without voltage being applied at the intermediate position, thereby yielding the electrical mobility diameter associated with 50% penetration through the HP-EMF. The capacity of the HP-EMF to distinguish among particles of different diameter can be reflected by its SSA, which is calculated from the slope (i.e. derivative) of the penetration curve with respect to mobility equivalent diameter at a penetration of 50%, with higher values indicating better sizing performance. SSA is defined as:


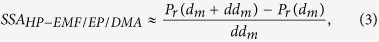


where *d*_*m*_ is electrical mobility diameter. Details related to computing the derivative in Equation (3) for the DMA are presented in the Supplementary Information.

Table 1 shows SSA values for the HP-EMF, EP and DMA. Evidently, the HP-EMF achieves a better SSA than the EP at all voltage settings and is of a comparable magnitude to that of the DMA, thereby providing a simple and cost-effective tool for deteminig the size of aerosol nanoparticles.

### Particle Tracking and Semi-Empirical Models

The analytic model described above provides simplified information regarding the performance of the HP-EMF. It only captures the electrostatic behaviour of the HP-EMF and does not consider the role of diffusion, which can be important in the decelerating region (i.e. Zone I) of the HP-EMF. To further investigate whether this model adequately captures the physical principles of the HP-EMF, we developed a particle tracking model which takes into account all relevant physical processes, including the role of diffusion. This model tracks nanoparticle trajectories by coupling their motion to the electrostatic and flow fields. The algorithm works by solving for the electric and fluid flow fields and then introducing equally spaced and singly-charged nanoparticles of a certain electrical mobility (and hence size) into the HP-EMF, followed by tracking their trajectories. Nanoparticles are tracked until they deposit within, or penetrate through the HP-EMF. With a time-step of Δ*t*, the axial and radial displacements are given respectively by:


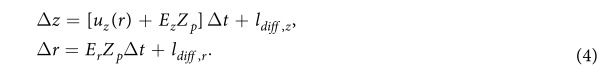


Here *u*_*z*_ is the axial (flow) velocity, *E*_*z*_ and *E*_*r*_ the axial and radial component of the electric field given respectively by 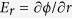 and 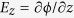, and *l*_*diff,z*_ and *l*_*diff,r*_ the axial and radial diffusional displacement of the particles, respectively, estimated from normal distributions with a mean at zero and a variance of 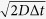, i.e. 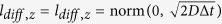, with *D* being the diffusion coefficient of the nanoparticles^20^.

Furthermore, we applied the semi-empirical model of Bezantakos *et al*.^21^ which captures both the electrostatic and diffusional behaviour of the HP-EMF, albeit with four adjustable parameters. The semi-empirical model is given by:


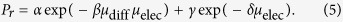


For laminar flow, 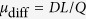 and 
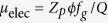
, with *Q* being the flow rate through the HP-EMF and *f*_*g*_ a factor that accounts for fringing effects in the electric field^21^.

The performance of all three modelling approaches against the measurements is shown in Fig. 4 when 1 kV was applied at the intermediate electrode, as this test setting measured nanoparticle diameters across a wide range from 1 to 55 nm. Analysis of covariance^22^ was used to test whether the analytical, nanoparticle tracking and semi-empirical models agreed with measured penetration data. The null hypothesis tested was that all three models yield the same slope with respect to measured data, which is equal to one in the case of perfect agreement. A two-tailed F test (numerator and denominator degrees of freedom of 2 and 27, respectively, F-value = 0.941, *p* = 0.403) showed that all three modelling techniques were able to reproduce the measurements. This was due to the slope of the predictions against the measurements having values spanning from 0.999 to 1.027 and hence being statistically equal to one. Consequently, it is possible to model the HP-EMF using techniques with varying levels of physical detail, whilst demonstrating statistical agreement with the measurements.

Even though the semi-empirical model is motivated by physical principles, the fact that four parameters need to be fitted to reproduce an experimental dataset does not make it a parsimonious approach for modelling the HP-EMF. In contrast, the analytic model (i.e. Equation 1) can contain one adjustable parameter which involves fitting the mean electric potential 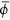 in Zone I (i.e. the decelerating segment). The reduction in potential for the analytic model reflects the lower electric potential that exists along the axis of symmetry in the HP-EMF. The adjustable potential in the analytic model makes physical sense, as Coulomb’s Law dictates an inverse square relationship for the electrostatic force with distance from a point charge, which also reduces the electric field strength and potential as the axis of symmetry is approached in the radial direction. Alternatively, calculating the electric potential analytically (i.e. using Equation 2) yields an estimated potential of 491 V along the axis of symmetry in Zone I when the potential applied to the surface of the HP-EMF is 1000 V. Relative to the fitted potential of 581 V (needed for fitting the analytic model to the measurements using the same conditions; i.e., 1000 V applied surface potential), this is a difference of approximately 15%. Substituting the electric field strength (calculated analytically) into the analytic model provides an accurate yet simple method for modelling the HP-EMF. The method, however, is still sufficiently simplistic since the role of diffusion is not accounted for. The good agreement between measurements and predictions by this method (i.e. using the analytically determined electric field given by Equation 1 in the analytic penetration model given by Equation 2) indicates that diffusional deposition in the decelerating zone is not an important mechanism in the HP-EMF for the flow rates and nanoparticle sizes investigated here. Apart from agreement with measurements, this modelling approach is validated by its excellent agreement with the nanoparticle tracking model, which captures all relevant physical phenomena, without the use of any adjustable parameters (i.e. the flow and electric fields are calculated analytically throughout the whole HP-EMF).

### Applications of the HP-EMF

The results discussed so far clearly show the effectiveness of the HP-EMF for nanoparticle sizing, as well as the agreement between measurements and existing theory. A key observation is that SSA improves for sub-10-nm nanoparticles, which is important for two main reasons. Firstly, by reducing the potential applied to the HP-EMF below 500 V, it should be possible to size-segregate ions and atomic clusters that are not easily amenable to analytical determination with existing aerosol instrumentation. Provided that such small clusters/nanoparticles are self-charged or can be efficiently charged (this is a remaining challenge in the field), it should be possible to measure their mobility spectra with the HP-EMF by scanning through a range of potential differences between the inlet/outlet and the intermediate electrode. Fields where nanoparticles smaller than 10 nm in diameter are of interest include nanotechnology, where the production of nanomaterials having characteristic sizes down to that of atomic clusters exhibit unique properties^23^,^24^, as well as new particle formation in the atmosphere, which is key to understanding the impacts of air pollution on human health and climate change^25^. By sizing nanoparticles in the sub-10-nm size range, the HP-EMF offers the capability to investigate such processes. Secondly, apart from measuring their size, the HP-EMF can probe size-dependent properties of nanoparticles in tandem with a DMA. This includes measuring nanoparticle properties such as volatility and hygroscopicity^26^, whereby the HP-EMF can be used to replace a DMA in complex experimental apparatuses. Using the HP-EMF in these systems will lead to greater portability and ease of use of such instruments in field and laboratory investigations.

Another potential application for the HP-EMF involves tunable filtration of nanoparticles by varying the applied voltage, as only those above a threshold diameter can be transmitted through it. There are many applications where nanoparticle filtration is required, such as providing clean air for homes, schools and office buildings^27^ as well as in vehicle exhausts^28^, whereas tunable filtration can find useful applications in nanoparticle and nanomaterial synthesis for various technological applications^23^.

It is widely acknowledged that low-cost sensor development is a critical requirement to improve understanding of nanoparticle impacts on humans, health and the environment^29^,^30^. As a step toward this need, we have developed, tested and modelled a HP-EMF that shows very promising agreement with existing theory, high SSA down to a few nanometres in size, as well as significant potential to spawn new measurement applications involving nanoparticle filtration and sizing. Furthermore, its applications are not just restricted to nanoscience and technology. As stated previously, it should be possible to size or filter molecular clusters with this technique, thereby opening up further scientific and technological opportunities.

## Methods

### Design Overview of the High-Pass Electrical Mobility Filter

**Figure 2:**
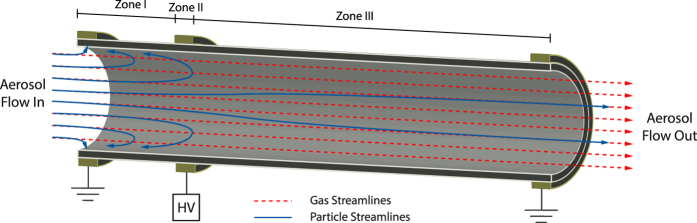
Schematic layout and nanoparticle trajectories for the HP-EMF.

**Figure 3:**
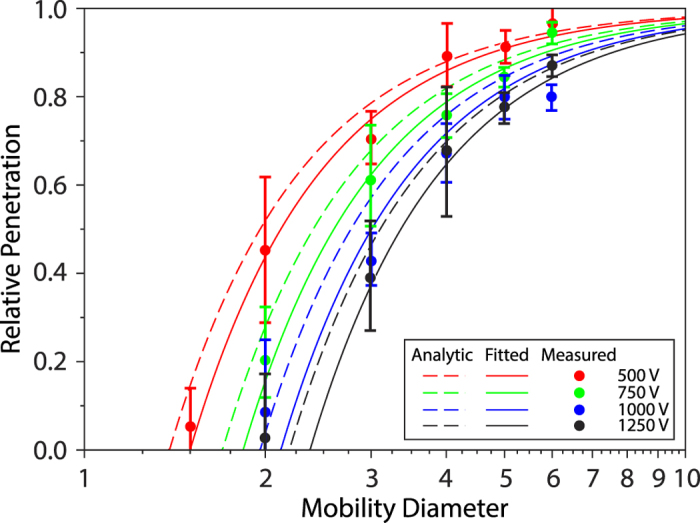
Experimental performance of the HP-EMF against the analytic model for particles having diameters in the 1–10 nm size range. Predictions from Equation 1 were fitted (solid lines) to the measurements using an electric potential in Zone I that was 58.1% of the value applied on the surface of the HP-EMF. When using the analytically determined electric potential (dashed lines; Equation 2) in the model gave good predictions of relative penetration compared with the measurements without any adjustment of parameters. Error bars indicate the range of one standard deviation of repeated measurements.

**Figure 4:**
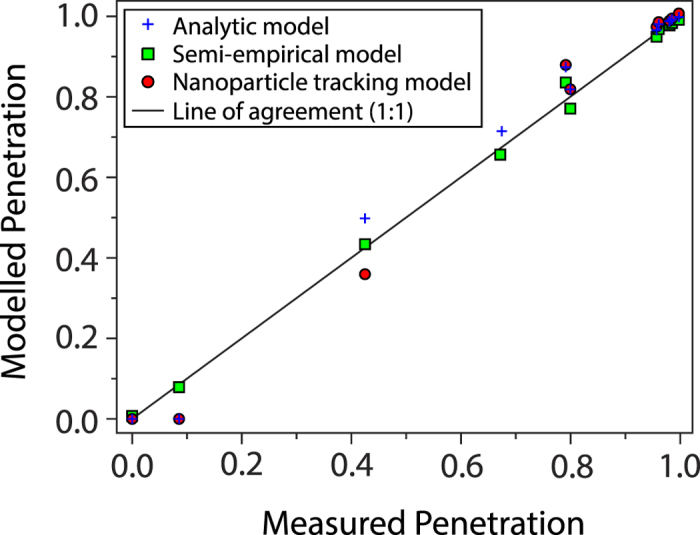
Performance of the HP-EMF at the 1 kV setting compared with three models over the nanoparticle size range of 1–55 nm. Parameter values fitted for the semi-empirical model were *α* = 0.3988, *β* = 117.8, *γ* = 0.5949, *δ* = 1.468.

A basic description of the physical principles underpinning the HP-EMF is provided here; however, a more detailed overview is provided by Bezantakos *et al*.^21^. The HP-EMF was constructed from Electrostatic Dissipative Material (EDM; Freelin Wade, Model 1A-405-81) with a length of 240 mm, an inner diameter of 6.4 mm and a wall thickness of 1.65 mm. Three metallic rings were placed along the HP-EMF; one at the inlet, one at the outlet and the other at an intermediate position (40 mm downstream of the inlet). The inlet and outlet were grounded and high voltage (or no voltage) was applied at the intermediate position. In testing, the HP-EMF was placed downstream of a DMA which transmitted monodisperse positively charged nanoparticles. A key feature of the HP-EMF is that the incoming nanoparticles have the same polarity as the high voltage power supply (i.e. positive in our case). This design feature leads to the development of a decelerating segment (i.e. Zone I, Fig. 2) upstream of the intermediate location on the HP-EMF where high voltage was applied, and an accelerating segment downstream of it (i.e. Zone III). By varying the applied potential at the intermediate location, it is possible to ‘tune’ the penetration of nanoparticles through the HP-EMF as a function of their diameter. In its current configuration and operating conditions, good SSA is achieved with the HP-EMF for nanoparticles less than 30 nm in diameter. The size of a nanoparticle is inferred from measurements of relative penetration (*P*_*r*_) at a potential of *ϕ*, which is the particle number concentration (*N*) at the HP-EMF outlet when voltage is applied to it, divided by *N* when no potential is applied (i.e. 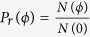).

### Overview of Measurements

Nanoparticles with different sizes were generated using electrospray, hot-wire and atomisation techniques. Each generation technique produced polydisperse nanoparticles, from which a monodisperse sample, consisting of positively charged nanoparticles, was obtained using two different DMA types. A high resolution differential mobility analyser (half-mini DMA^31^; SEADM) classified sub-10-nm nanoparticles and a custom-made cylindrical DMA classified nanoparticles greater than 10 nm in diameter. Nanoparticles were introduced at the inlet of the HP-EMF which was operated with and without an electric potential applied at the intermediate location. Relative penetration measurements were then made downstream of the HP-EMF with an ultrafine Condensation Particle Counter (CPC; TSI 3025A^32^) by making differential measurements of *N* with and without high voltage being applied.

In the first set of experiments, nanoparticles with a diameter of 1.5 nm were generated by electrospraying tetra-hexyl ammonium bromide salt (Sigma Aldrich, USA; 252816) with acetonitrile (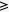
99.9% purity; Sigma Aldrich, USA; 34998) as a solvent. Nanoparticles between 2 and 6 nm in diameter were generated using a hot-wire generator with a Nickel filament^33^. In the second set of experiments, nanoparticles between 10 and 55 nm in mobility diameter were made by atomising ammonium sulphate (99.5% purity; Merck, Germany; 7783-20-2) using a TSI 3076 constant output atomiser. A solution of 0.025% ammonium sulphate (w/v) was prepared using ultrapure water (conductivity ≤ 0.055 *μ*S/cm). The flask of the atomiser was rinsed three times with ultrapure water before preparing a new solution. N_2_ (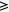
99.999% purity; Linde) was used as a carrier gas for all nanoparticle generation techniques.

In both sets of measurements, the HP-EMF was placed downstream of a DMA (half-mini DMA in the first set of measurements and custom-made DMA in the second set) and upstream of an ultrafine CPC. In the first set of measurements, the CPC was operated in high-flow mode (1.5 l/min) with modified saturator and condenser temperatures (41 °C and 8 °C, respectively) in order to count nanoparticles having diameters down to approximately 1 nm downstream of the HP-EMF^34^. After modifying (and stabilising) the CPC temperatures, checks were performed without a nanoparticle source to ensure that homogeneous nucleation was not occurring. In the second set of measurements, nanoparticles were counted with the ultrafine CPC operated at the default saturator and condenser temperatures (37 °C and 10 °C, respectively) in high-flow mode.

In both sets of measurements, the HP-EMF was tested with a range of potentials being applied to the tube in an alternating fashion. This entailed measuring *N* downstream of the HP-EMF with the applied potential alternating between 0 V and the required high voltage setting. A pausing period was used in between the zero and high-voltage settings to eliminate transient effects due to either increasing or decreasing the applied voltage during the measurement cycle. The time spent at each part of the measurement cycle was at least 45 s, which was sufficient for clearing nanoparticles before testing at the next set-point. Relative penetration was measured by taking the mean of *N* at the applied voltage, divided by that when the system was grounded. Only data with relatively stationary *N* fluctuations (±10%) were used for determining penetration.

### Overview of Nanoparticle Tracking Simulation

A Mersenne Twister generator^35^ was used to derive random diffusive displacements with a mean of zero and a standard deviation of 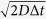. Simulations were conducted by tracking the trajectories of 2000 diffusing nanoparticles. Since the particular solution for the electric potential in Equation (2) is a series solution (in terms of eigenfunctions), 500 terms were used to compute the analytic solution on a mesh of 512 nodes in the radial direction and 960 in the axial. The same spatial mesh was used to calculate the flow field. A time step of 1 millisecond was used in the simulations. To generate predictions of relative penetration, simulations were first conducted with no potential applied to the HP-EMF, capturing nanoparticle motion driven by advection and diffusion only. These simulations accounted for diffusional losses in the tube, whereby some nanoparticles do not reach the HP-EMF outlet. In the second stage, a potential was applied to the HP-EMF to incorporate the effect of electrostatic deposition in addition to diffusion. The number of penetrating nanoparticles determined in the second stage divided by that in the first stage yields relative penetration *P*_*r*_.

## Additional Information

**How to cite this article:** Surawski, N. C. *et al*. A tunable high-pass filter for simple and inexpensive size-segregation of sub-10-nm particles. *Sci. Rep.* **7**, 45678; doi: 10.1038/srep45678 (2017).

**Publisher's note:** Springer Nature remains neutral with regard to jurisdictional claims in published maps and institutional affiliations.

## Supplementary Material

Supplementary Information

## Figures and Tables

**Figure 1:**
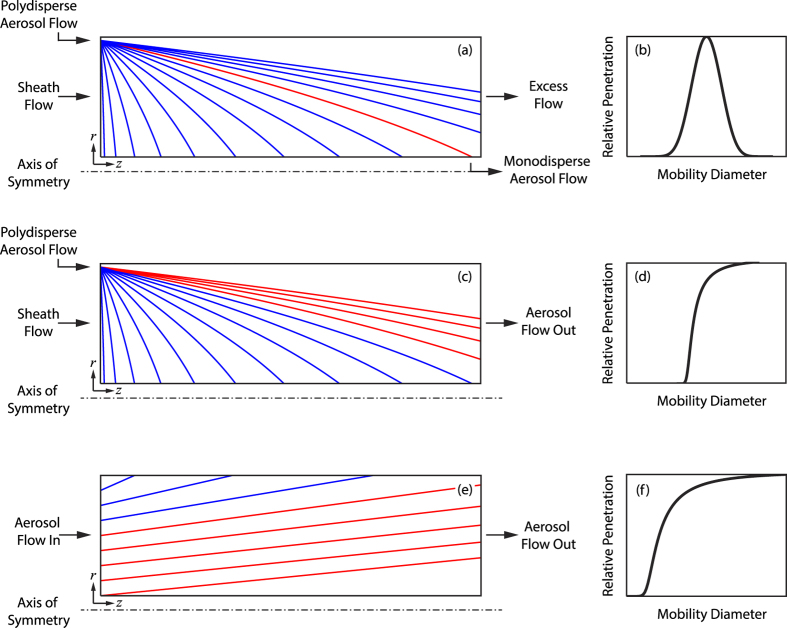
Operating principles of the Differential Mobility Analyser (DMA) the Electrical Aerosol Analyser (EAA) and a parallel plate electrostatic precipitator (EP). (**a**) Nanoparticle trajectories within the DMA. (**b**) Penetration curve of the DMA. (**c**) Nanoparticle trajectories within the EAA. (**d**) Penetration curve of the EAA. (**e**) Nanoparticle trajectories within a cylindrical EP. (**f**) Penetration curve of the EP. Blue and red lines indicate trajectories of nanoparticles that deposit within or penetrate each device in the outlet flow, respectively.

**Table 1:** Comparison of the SSA (nm^−1^) of the HP-EMF, an EP and a DMA.

HP-EMF Voltage	HP-EMF SSA	EP Voltage	EP SSA	DMA Voltage	DMA SSA
500	0.461	2.590	0.323	0.940	0.928
750	0.388	3.172	0.268	1.410	0.757
1000	0.319	3.663	0.225	1.879	0.657
1250	0.302	4.095	0.207	2.348	0.587

SSA is the slope of penetration with respect to mobility equivalent diameter at the point of 50% penetration, with higher values indicating better sizing performance. SSAs have been divided by 10^9^ for display purposes.
