# Supplementary material for: A tunable high-pass filter for simple and inexpensive size-segregation of sub-10-nm nanoparticles
Source: Sci Rep. 2017 Apr 27;7:45678. doi: 10.1038/srep45678 (PMC5406831; doi:10.1038/srep45678)
Supplement: Supplementary Information [file srep45678-s1.pdf]

Supplementary Information

A tunable high-pass filter for simple and inexpensive size-segregation of sub-10-nm nanoparticles

N. C. Surawski[1,†], S. Bezantakos[1], K. Barmpounis[1,2], M. C. Dallaston[3], A. Schmidt-Ott[2], and G. Biskos[1,2,*]

[1]Energy, Environment and Water Research Center, The Cyprus Institute, Nicosia, 2121, Cyprus

[†]Current address: School of Civil and Environmental Engineering, University of Technology Sydney, Ultimo, 2007, Australia

[2]Faculty of Applied Sciences, Delft University of Technology, Delft, 2628-CN, The Netherlands

[3]Department of Chemical Engineering, Imperial College London, London, SW7 2AZ, United Kingdom

[*]Corresponding author. Tel.: +357 22 208 618; +31 1527 88207.
E-mail: g.biskos@cyi.ac.cy; g.biskos@tudelft.nl.

Analytic calculation of the HP-EMF electric potential

Assuming that space and image charges are negligible, and that the field is axisymmetric, the electric potential ϕ can be calculated using Laplace's equation for a cylinder of radius R and length L:

$$\frac{1}{r}\frac{\partial}{\partial r}(r\frac{\partial \phi}{\partial r}) + \frac{\partial^2 \phi}{\partial z^2} = 0, \quad 0 \leq r \leq R, \quad 0 \leq z \leq L. \tag{1}$$

Boundary conditions include zero potential at both ends of the cylinder, a high voltage region of 10 mm length on the surface of the cylinder, as well as linearly increasing potentials from either end of the cylinder to the high voltage zone on the cylinder surface (Figure S.1). These boundary conditions are given by:

$$\phi(r,0) = \phi(r,L) = 0, \tag{2}$$

$$\phi(R,z) = \begin{cases} \phi_{HV}\frac{z}{z_0} & \text{if } z \leq z_0, \\ \phi_{HV} & \text{if } z_0 < z \leq z_1, \\ \phi_{HV}(1 - \frac{z-z_1}{L-z_1}) & \text{if } z_1 < z \leq L. \end{cases} \tag{3}$$

where ϕ_{HV} is the electric potential applied to the surface of the HP-EMF in the high voltage zone (i.e. zone II).

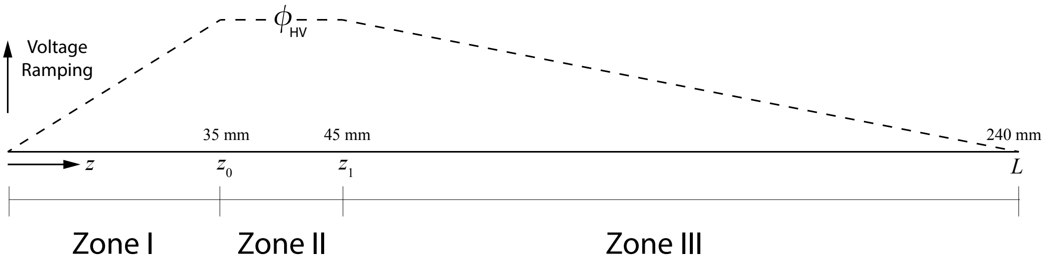

Figure S.1: Schematic of how voltage varies along the HP-EMF. The high-voltage zone spans the interval from z_0 to z_1, with potentials that increase linearly from zero at both ends of the HP-EMF.

An analytic solution to Equation (1) is possible via separation of variables by assuming that the solution is of the form $\phi(r,z) = V(r)W(z)$. Substituting this function

into Equation (1) yields the following system of two ordinary differential equations:

$$\begin{aligned}\frac{d^2W}{dz^2}+\lambda^2W=0,\\ \frac{d}{dr}r\frac{dV}{dr}-\lambda^2V=0.\end{aligned}\tag{4}$$

The sign convention for the separation constant λ^2 in Equation (4) is based on having homogeneous boundary conditions in the axial direction for this Sturm-Liouville problem. The general solution to the equations in (4) are:

$$\begin{aligned}W(z)=A\sin(\lambda z)+B\cos(\lambda z),\\ V(r)=CI_0(\lambda r)+DK_0(\lambda r).\end{aligned}\tag{5}$$

To satisfy the boundary conditions in Equation (2), we must have $B=0$ and $\lambda_m=\frac{m\pi}{L}$, where m is an integer. In addition, for the potential to remain finite as r approaches zero, we must have $D=0$. Therefore, the general solution to Equation (1) is:

$$\phi(r,z)=\sum_{m=1}^{\infty}C_m\sin(\lambda_m z)I_0(\lambda_m r),\tag{6}$$

where I_0 denotes a modified Bessel function of the first kind. The particular solution to Equation (1), can be obtained by finding values of C_m in Equation (6) that satisfy the boundary conditions on the surface of the cylinder given by Equation (3). Noting that z_0 and z_1 represent the start and end of the high voltage ring, respectively, the particular solution is:

$$\phi(r,z)=2\sum_{m=1}^{\infty}[\frac{\phi_{HV}L}{m^2\pi^2z_0}\sin(\frac{m\pi z_0}{L})+\frac{\phi_{HV}L}{m^2\pi^2(L-z_1)}\sin(\frac{m\pi z_1}{L})]\sin(\frac{m\pi z}{L})\frac{I_0(\frac{m\pi r}{L})}{I_0(\frac{m\pi R}{L})}.\tag{7}$$

Size Segregating Ability (SSA)

Penetration curves for the HP-EMF and EP are in a form that readily enables SSA to be calculated as they are both monotonically increasing functions. For the DMA, its transfer function needs to be converted into a cumulative distribution from which the derivative in the definition of SSA (see Equation 3 in main text) can be computed. For diffusing nanoparticles and balanced flows, the cumulative DMA transfer function $\Omega_c(Z_p)$, which is a function of electrical mobility, is found by integrating its transfer

function[1] with respect to Z_p, via:

$$\Omega_c(Z_p) = \int \frac{\sigma}{\sqrt{2}\beta}[\varepsilon(\frac{Z_p-(1+\beta)}{\sqrt{2}\sigma}) + \varepsilon(\frac{Z_p-(1-\beta)}{\sqrt{2}\sigma}) - 2\varepsilon(\frac{Z_p-1}{\sqrt{2}\sigma})]dZ_p. \quad (8)$$

Here $\varepsilon(x) = x\text{erf}(x) + \frac{1}{\sqrt{\pi}}e^{-x^2}$, erf(x)$=\frac{2}{\sqrt{\pi}}\int_0^x e^{-u^2}du$, u is a dummy variable of integration, β is an aerosol-to-sheath flow parameter and σ is a dimensionless diffusional spread parameter, given by $\sigma = \sqrt{0.081/\phi}$[1], assuming singly charged nanoparticles. Electrical mobility can then be transformed into electrical mobility diameter through the following equation[2]:

$$Z_p = \frac{neC_c}{3\pi\eta d_m}, \quad (9)$$

where n is the number of elementary charges, e is the electron charge, C_c is the Cunningham slip correction factor and η is the dynamic viscosity of air.

A relationship now exists between the cumulative DMA transfer function and electrical mobility diameter enabling computation of the derivative that defines SSA. Geometry parameters for a TSI 3071 DMA, with a flow temperature of 300 K and $\beta = 0.1$ (which is a typical value and was used in our measurements) was used to calculate SSA.

References

[1] M. R. Stolzenburg. *An ultrafine aerosol size distribution measuring system.* PhD thesis, University of Minnesota, 1988.

[2] W. C. Hinds. *Aerosol Technology: Properties, Behavior, and Measurement of Airborne Particles.* John Wiley & Sons, Inc., 2nd edition, 1999.
